# Detection of lost calamus challenges identity of isolated *Archaeopteryx* feather

**DOI:** 10.1038/s41598-018-37343-7

**Published:** 2019-02-04

**Authors:** Thomas G. Kaye, Michael Pittman, Gerald Mayr, Daniela Schwarz, Xing Xu

**Affiliations:** 1 Foundation for Scientific Advancement, Sierra Vista, Arizona 85650 United States of America; 2 Vertebrate Palaeontology Laboratory, Department of Earth Sciences, University of Hong Kong, Pokfulam, Hong Kong China; 3 Senckenberg Research Institute and Natural History Museum Frankfurt, Ornithological Section, Senckenberganlage 25, D-60325 Frankfurt am Main, Germany; 4 Museum für Naturkunde, Leibniz Institute for Evolution and Biodiversity Science, 10115 Berlin, Germany; 5 Key Laboratory of Vertebrate Evolution and Human Origins of Chinese Academy of Sciences, Institute of Vertebrate Paleontology and Paleoanthropology, Chinese Academy of Sciences, Beijing, 100044 China

## Abstract

In 1862, a fossil feather from the Solnhofen quarries was described as the holotype of the iconic *Archaeopteryx lithographica*. The isolated feather’s identification has been problematic, and the fossil was considered either a primary, secondary or, most recently, a primary covert. The specimen is surrounded by the ‘mystery of the missing quill’. The calamus described in the original paper is unseen today, even under x-ray fluorescence and UV imaging, challenging its original existence. We answer this question using Laser-Stimulated Fluorescence (LSF) through the recovery of the geochemical halo from the original calamus matching the published description. Our study therefore shows that new techniques applied to well-studied iconic fossils can still provide valuable insights. The morphology of the complete feather excludes it as a primary, secondary or tail feather of *Archaeopteryx*. However, it could be a covert or a contour feather, especially since the latter are not well known in *Archaeopteryx*. The possibility remains that it stems from a different feathered dinosaur that lived in the Solnhofen Archipelago. The most recent analysis of the isolated feather considers it to be a primary covert. If this is the case, it lacks a distinct s-shaped centerline found in modern primary coverts that appears to be documented here for the first time.

## Introduction

Arguably one of the best known and most iconic of fossil vertebrates, specimens of the “urvogel” *Archaeopteryx* have been found for more than a century in the Solnhofen limestones of Southern Germany^1^. As the first feather fossil ever discovered^1,2^, the isolated feather long rivaled the London specimen as the holotype of *Archaeopteryx lithographica*, before the latter was eventually designated as a neotype^3^. This fossil is represented by two slabs, which are in the collections of museums in Berlin and Munich, respectively. The known specimens of *Archaeopteryx* (11 or 12: the urvogel identity of one specimen has recently been challenged^4,5^) include some with feathers preserved as limestone impressions. This is contrasted by the isolated feather, which has a dark coloration and preserves as a film of carbon^6–11^ or manganese dioxide^1^. Most notably, the specimen has been characterized by the mystery of the “missing quill” - the originally reported calamus is today invisible in the fossil^10^.

**Figure 1:**
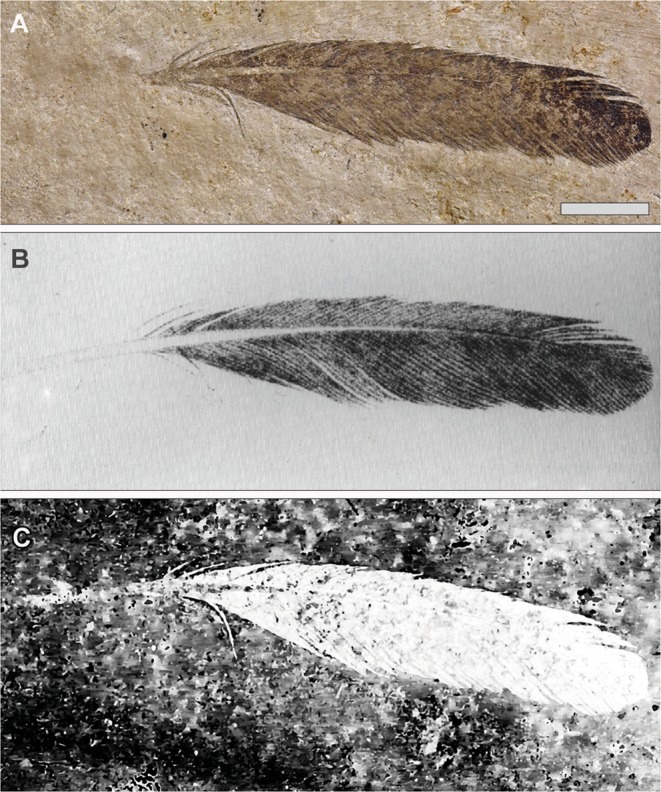
The isolated *Archaeopteryx* feather, Berlin specimen MB.Av.100. (**A**) As it looks today under white light (see Plates 1 & 5 [Fig. 1] of^7^, Fig. 1A of^8^ and Plate 10 of^14^). (**B**) Original drawing from 1862 by von Meyer^2^. (**C**) Laser-Stimulated Fluorescence (LSF) showing the halo of the missing calamus (negative image). See Fig. S2 for additional images of the main slab, specimen BSP 1869 VIII 1 (‘Munich slab’). Scale bar 1 cm.

**Figure 2:**
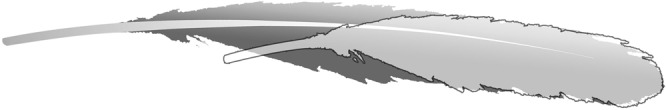
Overlay of the isolated feather MB.Av.100 scaled to the same size as the most similar secondary feather in the wing of the Berlin *Archaeopteryx* MB.Av.101. Significant foreshortening of the isolated feather does not support its association with *Archaeopteryx*.

Previous analyses of the isolated feather have been controversial, with disparate identifications as a primary (possibly a remicle of a larger specimen^7^; distal primary^12^), secondary^1,7^ (found as a distal secondary when compared to *Columba* and *Pica*^7^) and primary covert^8^. The lack of a preserved calamus added to the difficulty of the task. The calamus was first described and drawn in 1862^2^, but no obvious evidence of it remains today^10^ (Fig. 1). Possible explanations for the lack of a visible calamus on the more complete Berlin slab could be from damage incurred during past cleaning, re-preparation or handling of the slab (finger contact e.g. Fig. S2) as well as repeated exposure to daylight. However, there is no definitive evidence that attributes such damage to these particular sources. X-ray fluorescence^13^ and UV imaging studies of the feather did not report the missing quill (Figs 5, 6 of Plate 9 and Figs 1,2 of Plate 10 in^14^; Fig. 5.8 of^1^).

During an examination of the Berlin slab, a geochemical halo of the missing calamus was recovered for the first time using Laser-Stimulated Fluorescence (Fig. 1). This technique uses a high power laser to reveal geochemical differences in the specimen and matrix which fluoresce with different colors^15–17^ (see Materials and Methods). The length and width of the calamus halo matches that of the original published description^2^ (Fig. 1). Microscopic examination revealed past preparation had engraved around the outline of the feather and inadvertently prepared away the calamus at some unknown point in the past. Thus, the recovered geochemical halo is a chemical breakdown residue fluorescing immediately beneath the surface of the original carbon or manganese dioxide film.

The feathers are clearly defined in many *Archaeopteryx* skeletons^1^. The feather impressions from some of the more complete specimens allows for detailed morphologic measurements^1,18^. The general morphology of *Archaeopteryx* feathers is considered similar to modern birds, allowing cautious comparisons with living taxa^1^.

As in extant birds, the primaries of *Archaeopteryx* are characteristically straight and have vane asymmetry^19^. Their straightness does not match the isolated feather and they are also generally more asymmetrically vaned. The isolated feather’s identification as a primary feather has also been historically argued against^1,7^. *Archaeopteryx* lacks a bastard wing (alula)^1^, so the identification of the isolated feather as an alula feather of *Archaeopteryx* can be excluded.

The isolated feather is also not a tail feather (rectrix) of *Archaeopteryx*. The distal rectrices of *Archaeopteryx* are extremely long and symmetrical in outline at the tip (eleventh specimen: Fig. 2E of^18^), two features absent in the isolated feather. The isolated feather shares a general asymmetry in outline and rachis position with the lateral rectrices, but the curvature of the rachis is too severe in the isolated feather to form the frond pattern seen in *Archaeopteryx* (eleventh specimen: Fig. 2F of^18^). The tail feathers of the London specimen lack asymmetrical vanes, which also contrasts with the morphology of the isolated feather^1^.

The secondary feathers in the known *Archaeopteryx* specimens are the closest matches to the general feather outline of the isolated feather. Unfortunately, no other feathers stand alone in other *Archaeopteryx* specimens with feather preservation, but measurements of the isolated feather can be compared to the secondaries of the Berlin specimen, which preserves the most complete wing feathering of *Archaeopteryx*^1,14^. The outline of the isolated feather was superimposed onto a version scaled to match the width of the most similar secondary feather in the Berlin specimen (Fig. 2). This comparison reveals that the isolated feather is 1/3 shorter than required to scale to the secondaries of the Berlin *Archaeopteryx* wing. Unfortunately, the specimens larger than the Berlin specimen (London and Solnhofen) as well as the smallest urvogel (Eichstätt) both have poorly preserved feathering^1^, so this cannot be compared across ontogeny.

A range of secondary feather counts has previously been reconstructed along the ulna of the Berlin specimen (ten^20^, twelve^21^, fourteen (Fig. 6.18 of^1^) and twelve to fifteen^22^), but the reliability of these counts has been questioned^18^. Scaling the isolated feather to match the length and spatial overlap in the wing of the Berlin specimen (Fig. S7) shows that 7 secondaries could fit along the wing, significantly fewer feathers than past reconstructions. If the isolated feather was from a subadult as suggested by Wellnhofer^1^, then the feather count on the shorter ulna would be even less. As mentioned, this cannot be compared across ontogeny as the largest and smallest *Archaeopteryx* specimens (Solnhofen and Eichstätt) have poorly preserved feathering^1^. Nevertheless, these data raise questions about the fit of the isolated feather to the wing of *Archaeopteryx*.

**Figure 3:**
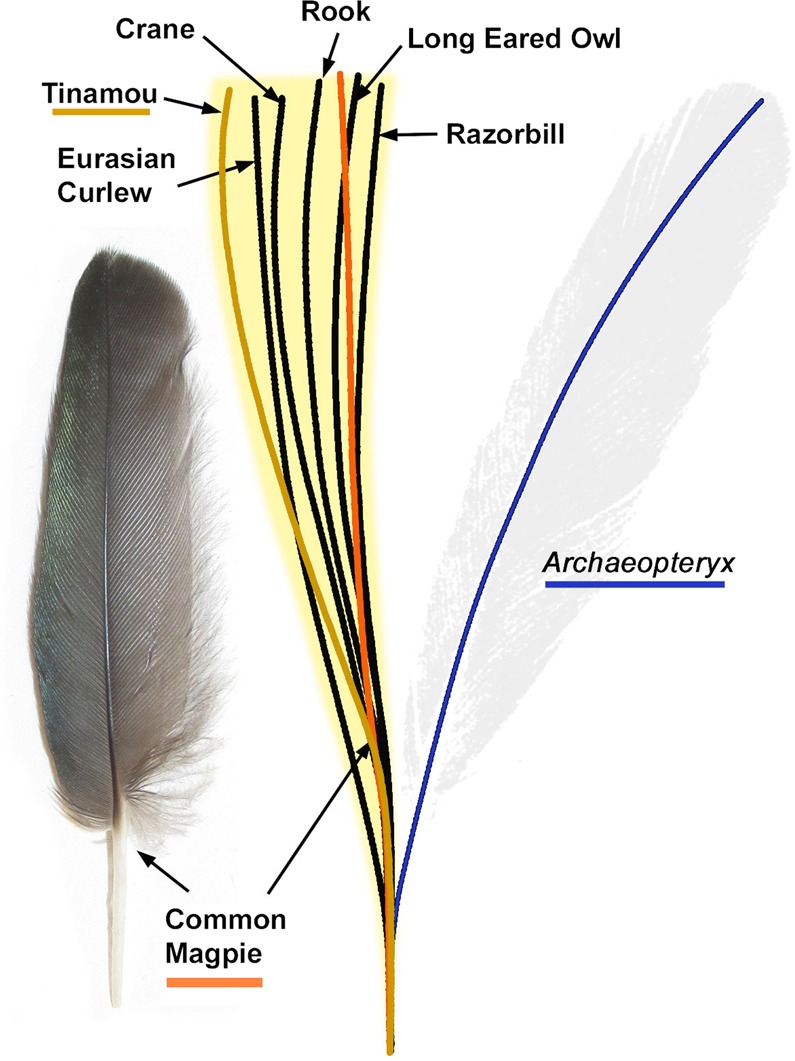
Size-normalized centerline calamus-rachis traces for the primary coverts of 24 modern birds compared to the trace of the isolated feather (Berlin specimen, MB.Av.100). The blue line is the isolated feather’s trace whilst the orange line is from the common magpie (*Pica pica*, Fig. S3) whose wing has been cited as the isolated feather’s closest modern match^1,7^. In brown is the centerline trace from a modern Undulated Tinamou (*Crypturellus undulatus* UWBM 71526, Fig. S4), which belongs to the only groups of extant palaeognaths with flight capabilities. The yellow zone represents the area covered by the traces of all 24 measured feathers, including a 1.5% error zone allowing for taphonomic flex (see Fig. S1). In all cases the isolated feathers centerline is a large departure from modern primary coverts.

The remaining possibilities for the isolated feather are as a covert or a contour feather. However, a determination is less straightforward. Little is known about the contour feathers of *Archaeopteryx*, although modern contour feathers typically have less robust calami than the isolated feather. As a covert, the isolated feather is very different to those of extant birds. In living birds, the secondary coverts attach to the calamus of the secondary flight feathers at an angle (Fig. S8). This configuration necessitates a shorter calamus than the primary coverts, which are in place alongside the primary feather calamus. The robust calamus of the isolated feather is therefore too large for a secondary covert, so this identification is not supported. The most recent analysis of the isolated feather considered it to be a primary covert^8^. The size-normalized calamus-rachis centerlines of primary coverts from 24 modern birds, including those of different body sizes, were compared to the isolated feather (Fig. 3). All possess a calamus-rachis centerline that curves towards the leading edge of the wing from the centerline of the calamus, unlike the rachis centerlines of the other feather types present in the same wing specimens^7,19,23,24^ (Figs 3, S3–S6). This ‘S-shaped’ centerline described here for the first time, appears to be a defining characteristic of primary coverts across a very broad range of modern species, including the palaeognath tinamou. In contrast, the centerline of the isolated Solnhofen feather curves strongly toward the wing’s trailing edge (see blue line in Fig. 3) so does not match the morphology of primary coverts in modern birds^7,19,23,24^.

In summary, the isolated feather is not conformal to known *Archaeopteryx* specimens as a primary, secondary or tail feather. Its preservation as a dark film also differentiates it from all other known specimens^1,6^. The isolated feather as argued here lacks any close morphological connection to the 11 or 12 known *Archaeopteryx* skeletons (see status of Haarlem specimen^4,5^), but not all feathers of *Archaeopteryx* are known. However, based on known feather preservation in *Archaeopteryx*, this study raises the possibility that the isolated feather may belong to another basal avialan or even a non-avialan pennaraptoran, increasing the low theropod diversity of the Solnhofen Archipelago^1,4,25–27^. This hypothesis would be in agreement with comments made in Opinion 2283 (Case 3390) of the ICZN Commission^3^ as well as the recent removal of the Haarlem specimen from *Archaeopteryx*^4^. The feather remains an enigma so we caution against the isolated feather’s association with *Archaeopteryx*.

## Material and Methods

### *Archaeopteryx* specimens studied

1. The single feather: BSP 1869 VIII 1 (main slab, ‘Munich slab’), Bavarian State Collection of Paleontology and Geology, Munich; MB Av.100 (counterslab, ‘Berlin slab’), Museum für Naturkunde, Berlin, Germany.
2. London specimen: NHMUK 37001 (main slab), Natural History Museum, London, UK.
3. Berlin specimen: MB.Av.101 (main and counterslab), Museum für Naturkunde, Berlin, Germany.
4. Haarlem specimen: TM 6928 (main slab), Teylers Museum, Haarlem, Netherlands; TM 6929 (counterslab).
5. Eichstätt specimen: JM 2257 (main and counterslab), Jura Museum, Willibaldsburg, Germany.
6. Solnhofen specimen: BMMS 500 (main slab), Bürgermeister Müller Museum, Solnhofen, Germany.
7. Munich specimen: BSP 1999 I 50 (main and counterslab), Bayerische Staatssammlung für Paläontologie und Geologie, Munich, Germany.
8. Daiting specimen: unknown specimen number, unknown current repository details.
9. Bürgermeister Müller (‘chicken wing’) specimen: unknown specimen number, on permanent loan to Bürgermeister Müller Museum by the families Ottmann and Steil.
10. Thermopolis specimen: WDC CSG 100 (main slab). Wyoming Dinosaur Center, Thermopolis, USA.
11. Eleventh specimen: no. 02923 on the register of cultural objects of national importance of Germany (Verzeichnis national wertvollen Kulturgutes), on long-term loan to Bürgermeister Müller Museum.

### Modern bird specimens studied

*Museum collections.*

1. Tinamou (*Crypturellus undulatus*) - UWBM 71526; University of Washington Burke Museum of Natural History and Culture, Seattle, USA) (Fig. S4).
2. Common magpie (*Pica pica*) - FSA2016-01; Foundation for Scientific Advancement, Sierra Vista, USA).
  *Atlas of avian feathers at* www.vogelfedern.de/index-e.htm.
3. Common Crane *Grus grus*.
4. Tundra Swan *Cygnus columbianus*.
5. Common Magpie *Pica pica* (Fig. S3).
  *Atlas of avian feathers at* www.michelklemann.nl/verensite/start/index.html.
6. Peregrine Falcon *Falco peregrinus*.
7. Tufted Duck *Aythya fuligula*.
8. Black Headed Gull *Larus ridibundus*, example 1.
9. Sparrowhawk *Accipiter nisus*, example 5.
10. Mallard *Anas platyrhynchos*, example 3.
11. Swift *Apus apus*, example 4.
12. Little Ringed Plover *Charadrius dubius*.
13. Skylark *Alauda arvensis*.
14. Hen Harrier *Circus cyaneus*.
15. Long Tailed Duck *Clangula hyemalis*.
16. Lilac Breasted Roller *Coracias caudatus*.
17. Quail *Coturnix coturnix*.
18. Long-eared Owl *Asio otus*.
19. Razorbill *Alca torda*, example 1 (Fig. S6).
20. Teal *Anas crecca*, example 1.
21. Two Barred Crossbill *Loxia leucoptera*, example 3.
22. Giant Kingfisher *Megaceryle maxima*.
23. Black Kite *Milvus migrans*.
24. Whimberel *Numenius phaeopus*.
25. Eurasian Curlew *Numenius arquata* (Fig. S5).
26. Rook *Corvus frugilegus*.

Laser-Stimulated Fluorescence (LSF) imaging was performed according to the protocol of Kaye *et al*.^15,17^ so only an abbreviated version is provided here. A 405 nanometer laser diode was used to fluoresce the specimen following standard laser safety protocol. Thirty second time exposed images were taken with a Nikon D810 camera and 425 nanometer blocking filter. Post processing (equalization, saturation and colour balance) was performed in Photoshop CS6.

Primary covert feather analysis was performed from photographs. These were sourced from museum collections and the Vogel Federn and Michel Klemann online feather atlases^23,24^. The feathers of the latter two collections were flat bed scanned (see Supplementary Materials for discussion of flattening-related feather taphonomy). Feather centerlines were overlaid in Photoshop CS6, all centerlines were scaled to the same length.

## Supplementary information

Supplementary Information
