# Supplementary material for: Detection of lost calamus challenges identity of isolated Archaeopteryx feather
Source: Sci Rep. 2019 Feb 4;9:1182. doi: 10.1038/s41598-018-37343-7 (PMC6362147; doi:10.1038/s41598-018-37343-7)
Supplement: Supplementary file 1 — Supplementary Information [file 41598_2018_37343_MOESM1_ESM.docx]

# Detection of lost calamus challenges identity of isolated *Archaeopteryx* feather

Thomas G. Kaye^1*^, Michael Pittman^2^, Gerald Mayr^3^, Daniela Schwarz^4^ & Xing Xu^5^

**Supplementary Information**

**Taphonomic Bias**

Since feathers start off as three dimensional structures and are taphonomically compressed into a two dimensional film, the question arises as to the extent of feather distortion in this process. An experiment was conducted using the third secondary, third secondary covert and third primary covert from a Magpie (*Pica pica*) as it is believed to be the closest modern match to *Archaeopteryx*^1,2^ (Fig. S1). The secondary with the greatest curvature was intentionally selected. The secondary covert represents the small end of the scale.

Feather calami were aligned with lines drawn on a sheet of paper. White dots near the center and end of the rachis were used as reference points. The total length of the feathers was measured from the proximal end of the calamus to the distal white dot. The curvature was measured as the perpendicular offset from the centerline to the white dots. The variables: feather length = L1 (3D), L2 (crushed), perpendicular distance for mid rachis = C1, C2, perpendicular distance for rachis tip = T1, T2. Formula to calculate offset: (T1/ L1) – (T2/L2) same for other calculations.

A sheet of glass was vertically lowered onto the feathers crushing them flat. Measurements were taken again and the difference in percentage offset calculated (Table S1). Results show that in the most extreme case of the smallest feather, the mid-rachis offset was less than 1.5% of its length. The rest of the specimens measured were typically less than 1%. This experiment demonstrates that the taphonomic distortion is likely very minimal and this is backed up by the largely intact barb morphology on the single feather. The feathers measured in the referenced feather atlases were mostly scanned flattened by the scanner lid and as this experiment shows, would still represent sufficiently accurate data for this study.


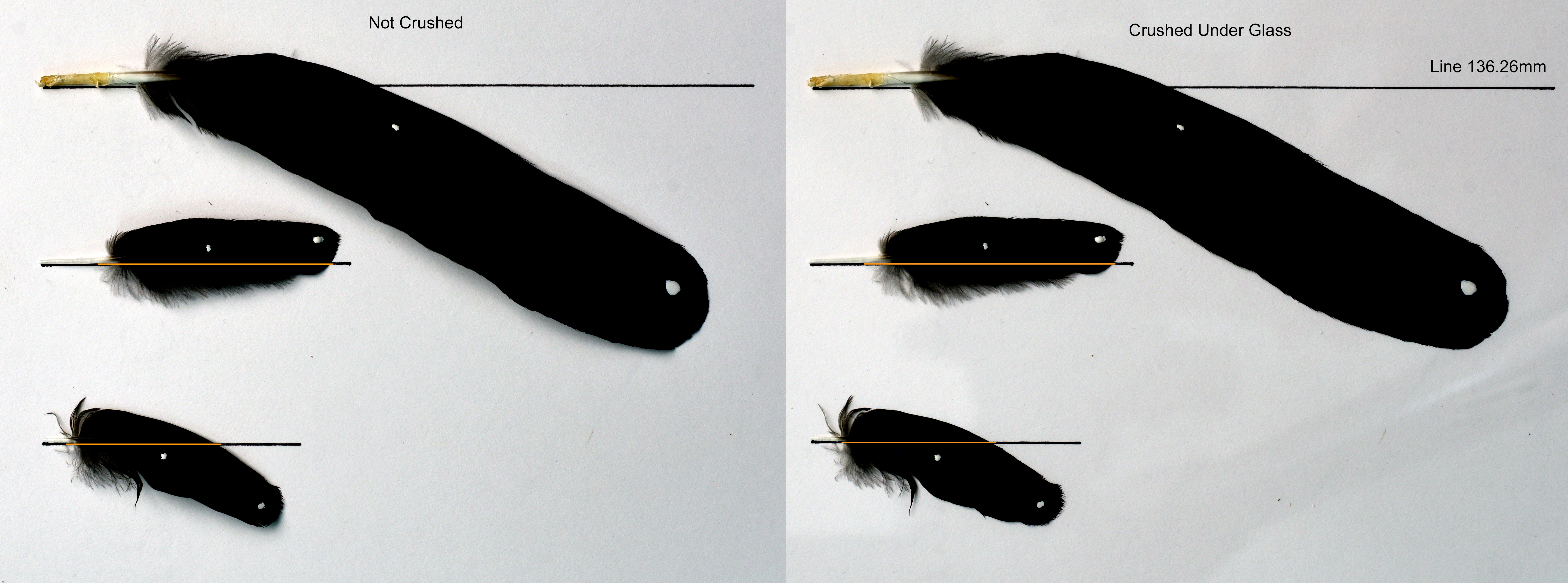


**Figure S1.** **Comparison of 3D and crushed magpie feathers.** After compression under glass, the offset from centerline was generally less than ±1.5%. This suggests that taphonomic processes are unlikely to cause severe distortions in the feather curvature.

| **Highest Curvature** | **Crush Difference** | **No Crush** | **Crush** |
| --- | --- | --- | --- |
| **3rd Secondary** |  |  |  |
| Crush Offset Rachis Tip % = | 0.04% | 28.90% | 28.86% |
| Crush Offset Mid Rachis % = | 0.19% | 5.98% | 5.79% |
|  |  |  |  |
| **3rd Primary Covert** |  |  |  |
| Crush Offset Rachis Tip % = | -0.10% | 8.35% | 8.45% |
| Crush Offset Mid Rachis % = | -0.64% | 5.48% | 6.12% |
|  |  |  |  |
| **3rd Secondary Covert** |  |  |  |
| Crush Offset Rachis Tip % = | 0.39% | 26.03% | 25.64% |
| Crush Offset Mid Rachis % = | -1.31% | 5.10% | 6.41% |
|  |  |  |  |

**Table S1.** **Distortion measurements for crushed and uncrushed feathers.**


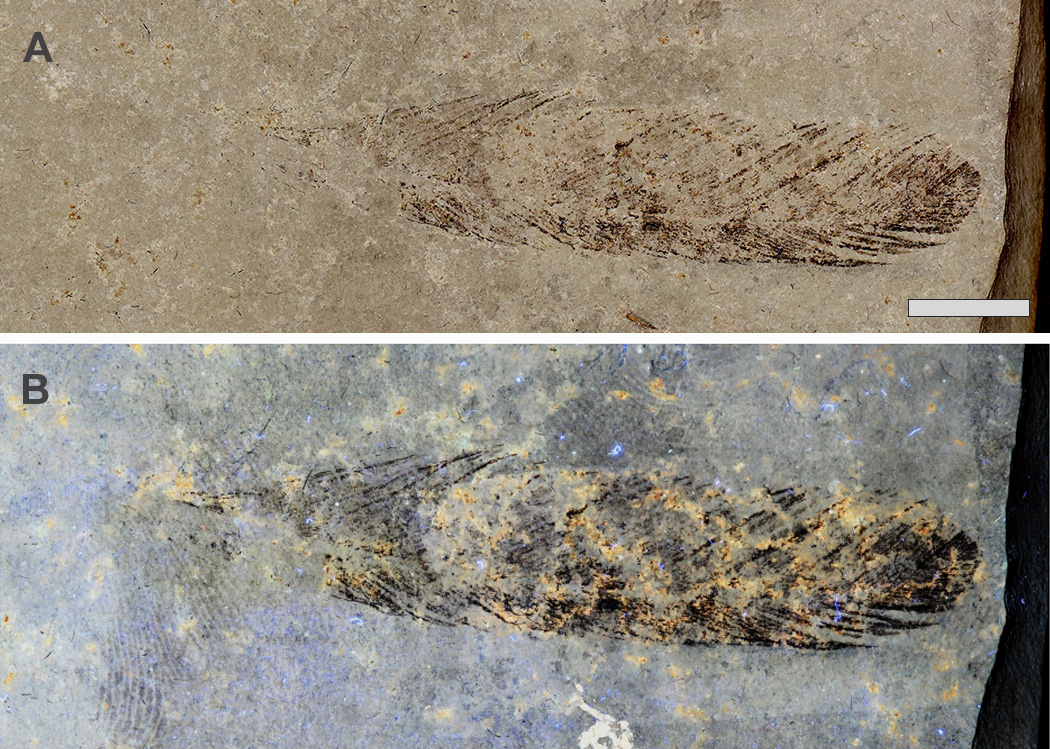
 **Figure S2.** **The main slab of the single feather BSP 1869 VIII 1 (‘Munich slab’).** **A.** White light image. **B.** Laser-Stimulated Fluorescence (LSF) image. Visible grey whorls are fingerprints. Scale bar 1cm.

**
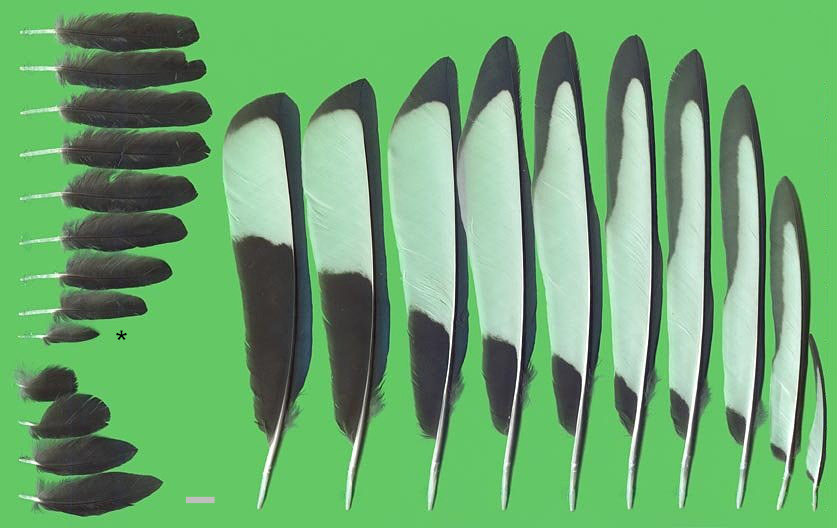
**

**Figure S3.** **Isolated feathers from the deconstructed wing of the Common Magpie *Pica pica*.** Primary coverts shown in the upper left. Distal covert marked with asterisk. Scale bar 1cm. Image used with the permission of Stephan Schubert and is hosted in the atlas of avian feathers at [www.vogelfedern.de/index-e.htm](http://www.vogelfedern.de/index-e.htm).


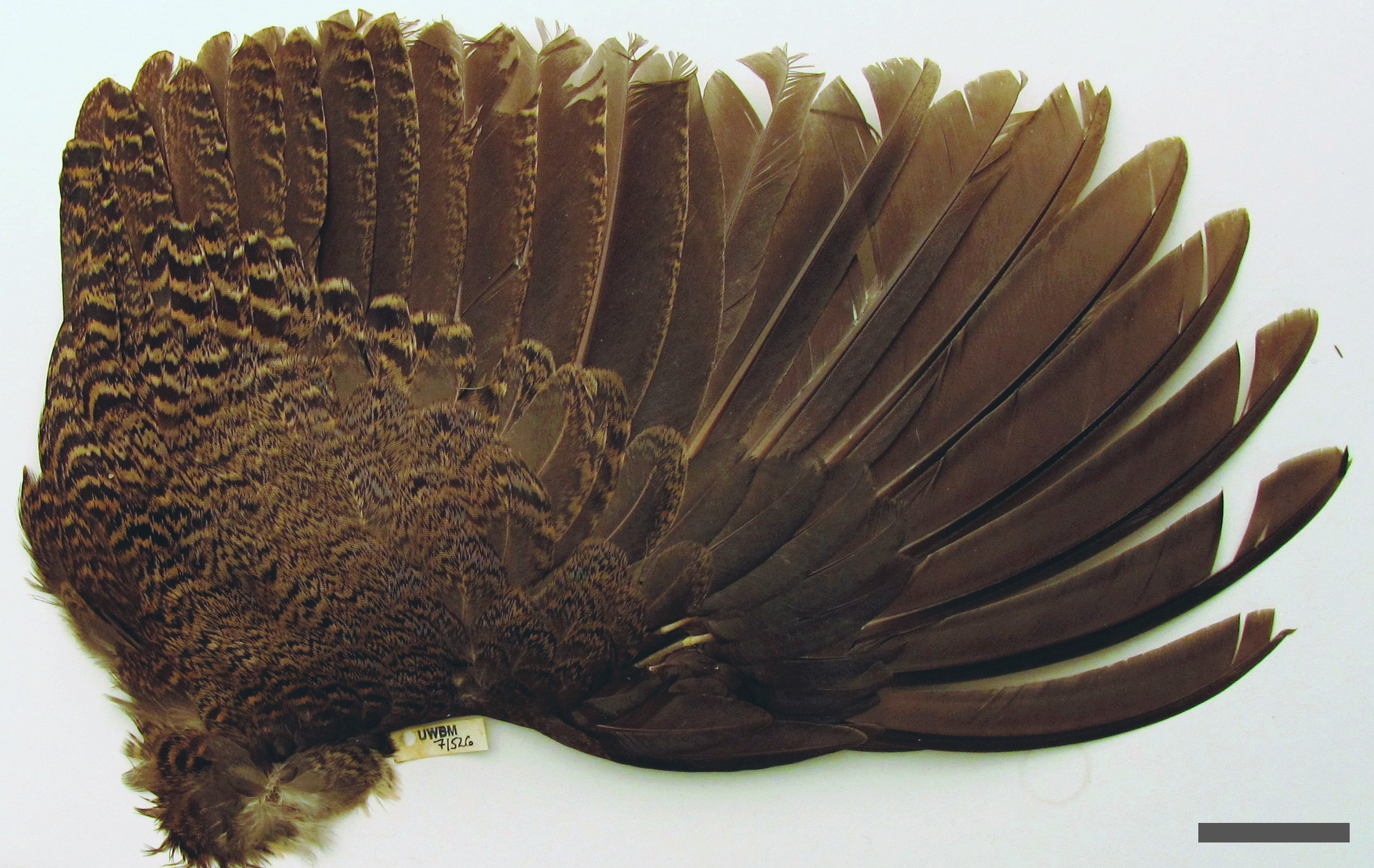
**Figure S4.** **Tinamou wing *Crypturellus undulatus* UWBM 71526.** Used in analysis shown in Fig. 2. Scale bar 3cm.

**
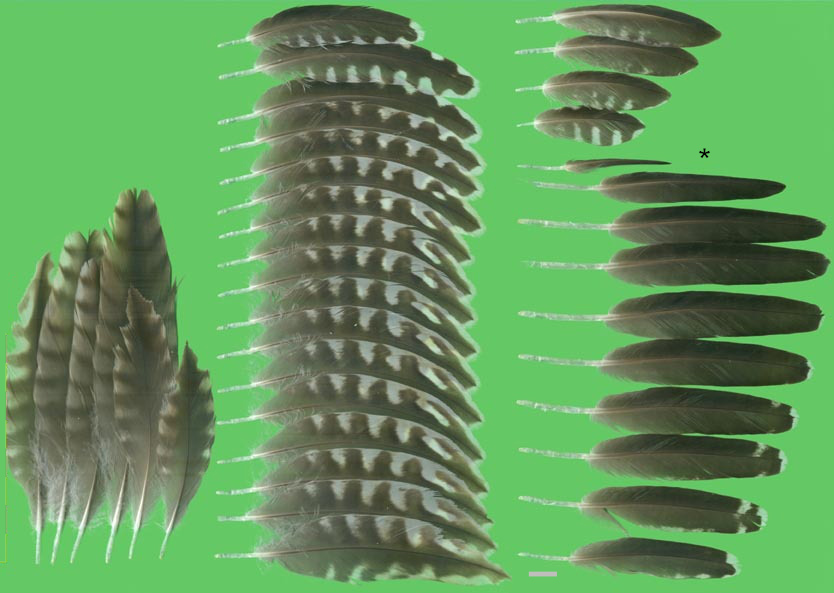
Figure S5.** **Isolated feathers from the deconstructed wing of the Eurasian curlew (*Numenius arquata*).** Primary coverts shown in the lower right. Distal covert marked with asterisk. Scale bar 1cm. Image used with the permission of Stephan Schubert and is hosted in the atlas of avian feathers at [www.vogelfedern.de/index-e.htm](http://www.vogelfedern.de/index-e.htm).

**
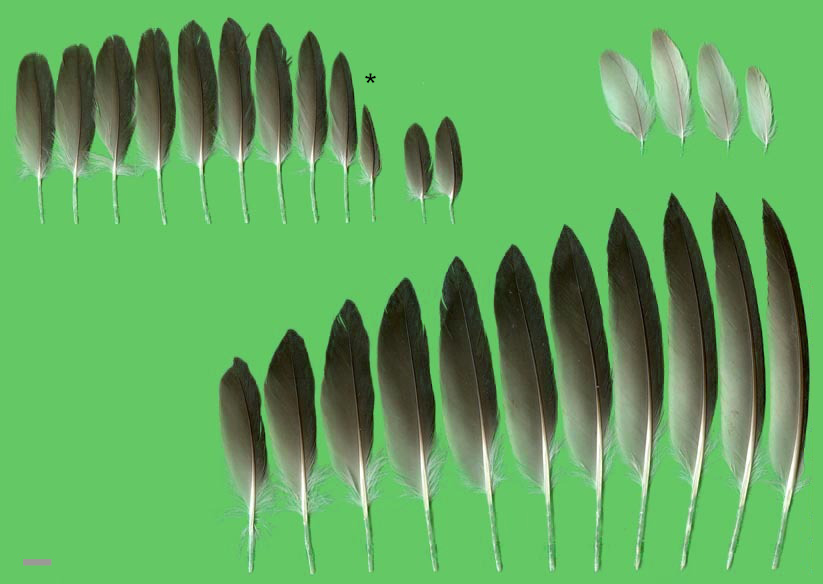
**

**Figure S6.** **Isolated feathers from the deconstructed wing of the Razorbill (*Alca torda*).** Primary coverts shown in the upper left. Distal covert marked with asterisk. Scale bar 1cm. Image used with the permission of Stephan Schubert and is hosted in the atlas of avian feathers at [www.vogelfedern.de/index-e.htm](http://www.vogelfedern.de/index-e.htm).


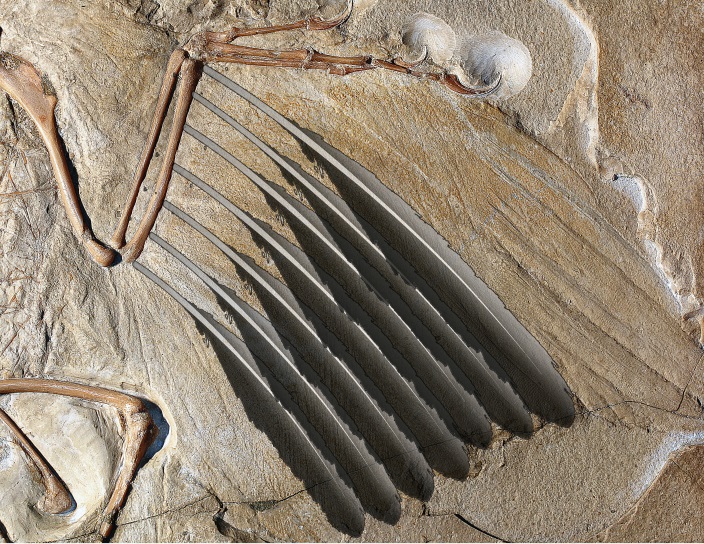


**Figure S7.** **The isolated feather scaled to match the lengths and overlap in the Berlin wing feathers (MB.Av.100).**


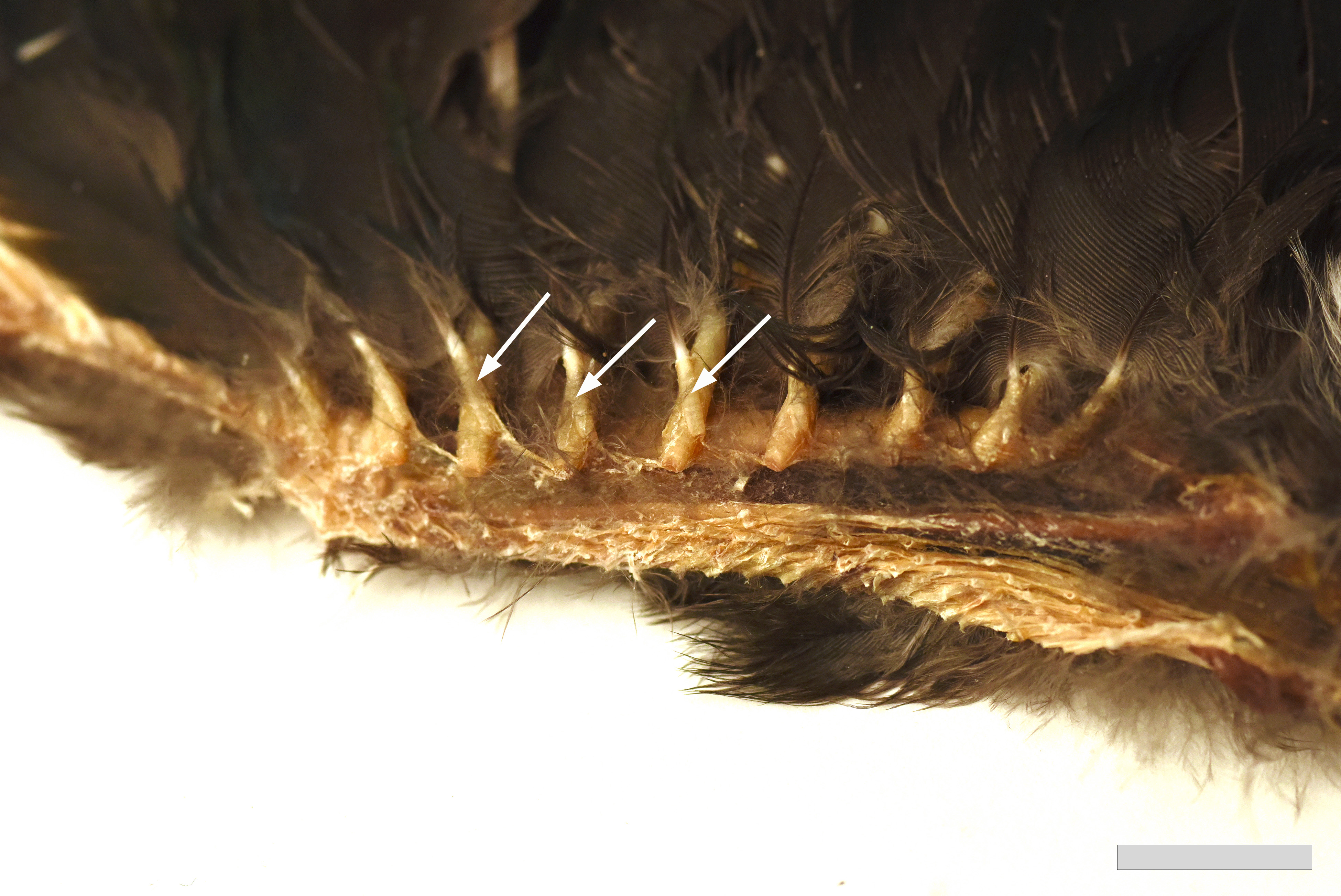


**Figure S8. Exposed wing of the common magpie (*Pica pica*, FSA2016-01).** Arrows show the attachment points of the secondary coverts to the secondary flight feathers. Scale bar 1cm.

**Additional Author Notes:** T.G.K, M.P and D.S. designed the project. All authors performed the research. T.G.K. & M.P. wrote the manuscript. All authors edited the manuscript.

**Supplementary References:**

1 Griffiths, P. J. The isolated *Archaeopteryx* feather (Die isolierte *Archaeopteryx-*feder). *Archaeopteryx* **14**, 1-26 (1996).

2 Wellnhofer, P. *Archaeopteryx - the icon of evolution*. (Verlag Dr. Friedrich Pfeil, 2009).
